# Research on the safety HME-CM approach of cranes in their service life cycle

**DOI:** 10.1371/journal.pone.0290830

**Published:** 2023-08-31

**Authors:** Ying Chen, Lianghai Jin

**Affiliations:** 1 College of Hydraulic & Environmental Engineering, China Three Gorges University, Yichang, 443002, China; 2 Hubei Key Laboratory of Hydropower Engineering Construction and Management, China Three Gorges University, Yichang, 443002, China; 3 Safety & Environment Science and Technology Ltd., Yichang, 443002, China; CINVESTAV IPN: Centro de Investigacion y de Estudios Avanzados del Instituto Politecnico Nacional, MEXICO

## Abstract

Crane is one of the vital components and lifelines of the construction industry. However, crane accidents happen regularly due to a variety of factors. Among them, the most far-reaching influence is the three factors of the human-machine-environment (HME). Moreover, how to carry out safety management for them is very critical. Considering the barriers to the goal, this study applied configuration management (CM) to HME data change management in cranes’ service life cycle and proposed a HME-CM framework and approach. First, based on the original CM theory, baselines were developed to accommodate changes in HME configuration. Second, we discussed the evolving trajectory of HME configuration and determined the content and updates to the baseline. Third, a marking method based on data provenance theory was proposed to achieve data consistency. Finally, two search procedures were designed to perform the tracking and tracing of HME configuration. This paper contributes to expanding the application of CM theory in crane safety management, ensures the controllability and traceability of crane configuration changes, and provides a new perspective on crane safety.

## 1. Introduction

Cranes are critical elements in achieving productivity in construction and occupy an important position among construction equipment due to their indispensable capabilities in vertical and horizontal transportation [1,2]. However, they are arguably also the main generators of on-site safety hazards [3]. Once a crane accident occurs, severe damage may be incurred, which not only threatens the safety of workers but also causes immediate damage to machinery, equipment, and buildings [4–6]. According to China’s State Administration for Market Regulation, the number of crane accidents ranks among the top three in special equipment accidents all year round and even ranked first in 2018. Therefore, crane safety is always a hot topic in the construction industry [7].

Crane safety issues can be regarded as complex sociotechnical system problems [8], which can be influenced by various factors across multiple dimensions [7]. So far, factors involved in crane accidents have been extensively studied. Tam and Fung [9] investigated tower crane safety related to the understanding and degree of executing statutory requirements and non-statutory guidelines for the use of tower cranes in the Hong Kong construction industry. They discovered that inadequate training and practitioner fatigue are two primary causes of unsafe practices in tower crane operations. Raviv et al. [3] qualitatively analyzed 212 incident stories and found that technical failures are the most hazardous risk factors within the tower crane domain. Marquez et al. [5] discussed the failure of two cranes and the striking similarities in failure circumstances. These include errors in the identification and interpretation of previous symptoms, in the mitigation measures undertaken, and in the risks assumed by personnel due to lack of information and training. Lingard et al. [10] investigated the causes and contributors to crane safety incidents in the Australian construction industry, and a total of 77 causal or contributing factors were identified in the analysis.

The prior research has promoted the development of targeted preventive strategies for crane accidents [8]. However, how to carry out systematic safety management against those factors is still a persistent problem. We can conclude from these studies that cranes have the characteristics of a long life cycle, complex equipment, susceptibility to the environment, and close contact with construction personnel, so those factors can be summarized into three types: human-machine-environment (HME) [8,11]. During the long service life cycle, the status of the involved parts, the working environment, and construction personnel information are in a state of frequent change, and the management of relevant data is highly complex, resulting in information that is often incomplete, inaccurate, or unavailable [12]. The management of data documents is an essential part of safety management, which ensures the controllability and traceability of crane configuration changes, makes change control possible, and provides references for relevant safety and maintenance decisions. Therefore, it is essential to control the change of the crane’s HME data in the service life cycle.

Therefore, we introduced configuration management (CM) theory to HME data management and process management, which includes baseline construction, change management, tracking, and tracing realization [13]. It focuses on recording HME configuration in an immutable, accurate, timely, and traceable way, helping keep configuration changes under control and enabling trace changes to give measures, solutions, and interventions [5,14]. Also, the data provenance method is applied to the CM marking process, and search procedures are proposed for more comprehensive management of the HME configuration to enable crane construction to be carried out smoothly and safely.

## 2. Framework construction of HME-CM

### 2.1 Basics of CM

CM is an effective tool for systems engineering management and an important product of the development process of complex weapon systems [15], which can effectively manage the configuration or functional product data of complex products such as aircraft and rockets [16,17]. CM provides a reliable method to control the system products, processes, and related documentation for the entire product life cycle [18]. It ensures consistency and traceability of parts, documents, or data throughout the product life cycle from design to manufacturing, use, maintenance, and scrap.

Configuration items are the basic units of CM. The lowest-level configuration item is individual and can be part of a higher-level configuration item. It is designated at an appropriate level for documenting performance attributes and managing changes to those attributes. Configuration items may vary widely in complexity, size, and type. But either way, the configuration of a configuration item is documented and controlled [18].

**Fig 1:**
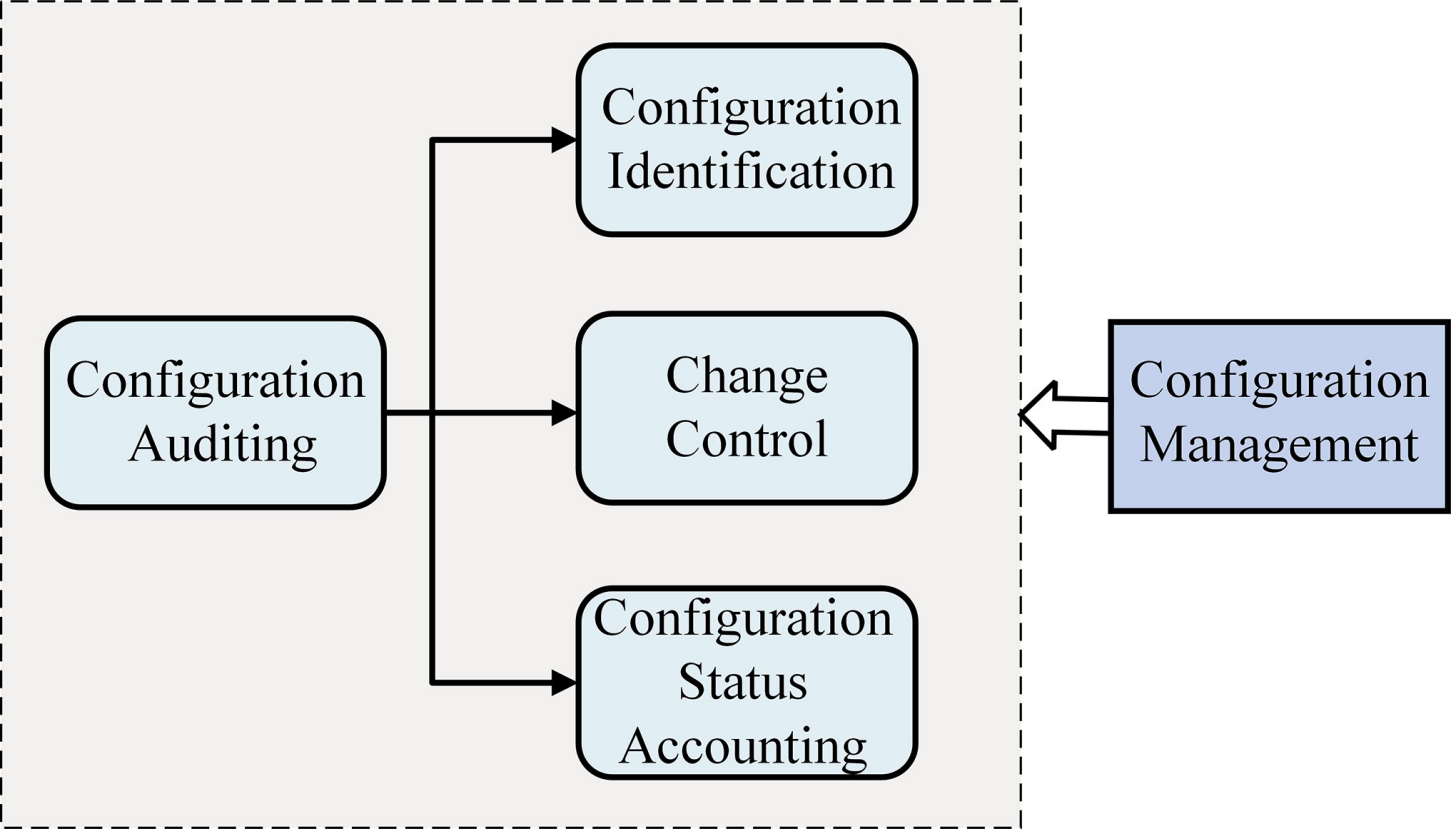
Configuration management process.

CM can be subdivided into four major processes: Configuration Identification, Change Control, Configuration Status Accounting, and Configuration Auditing [16,19]. The logical relationship between them is shown in Fig 1. The configuration identification process should ensure the establishment of initial configuration items and configuration baselines, then form the configuration documents, and make dynamic corrections after the project is carried out. Change control is at the heart of CM, which can achieve governance of changes and deviations of management objects through the control of configuration documents. Configuration Status Accounting and Configuration Auditing run through the entire product life cycle. Configuration Status Accounting is the record of the configuration items, project data, baseline status, etc. Configuration Auditing is the inspection to confirm that the product configuration conforms to its configuration document and the supervision and management of the Configuration Identification, Change Control, and Configuration Status Accounting process [15].

Different characteristics of various industries or products lead to the adaptation of CM and promote its theoretical expansion from the R&D design period to the full life cycle [13]. Meanwhile, data management using large digital data sets, along with rapid access to information, can provide the basis for more responsive, flexible, and real-time decision-making in projects [20]. Therefore, this paper attempts to combine the changes of HME-related data in the service life cycle of cranes with CM theory to explore an HME-CM framework and create baselines to conduct configuration identification and configuration change tracking [21].

### 2.2 Research object analysis

The service life of cranes in China is generally 15 to 30 years. During the entire service life, there are many construction personnel involved, as well as complex environmental changes, frequent mechanical maintenance, and support activities. All change activities may have an impact on their service life [22]. For maintenance activities only, the number of parts replacement nodes is huge, not to mention all the HME change nodes. HME-related data changes occur at various points in time throughout the crane’s service life. In order to ensure the safety of crane operation and quickly respond to emergency failures caused by humans, the environment, or the machine itself, it is necessary to keep abreast of the configuration of the crane and track the HME configuration data in real-time [23].

The entire life cycle of a crane can be further subdivided into a number of on-site service periods. Of course, in addition to the current repair and medium repair that will be performed at the construction site, in-plant overhaul activities may also occur between two on-site service periods.

The configuration changes in the service life are divided into three parts: human, machine, and environment. We regard a working personnel or a mechanical part as a configuration item, while the environment is relatively special. We regard the overall environment as a configuration item. Then, a human configuration item documents basic information such as registration, certification, safety training records, physical and mental health status assessments, history records of violation operations, etc. A machinery configuration item documents basic attributes, quality attributes, and location. And an environmental configuration item documents meteorological and foundation conditions. This configuration item information constitutes configuration documents.

At the beginning and end of each on-site service activity, it is necessary to clarify the current configuration status of the crane to ensure that the configuration change records are complete and detailed and to provide a basis for accident traceability and maintenance plan formulation. Meanwhile, to achieve the traceability of HME configuration, it is necessary to control the change process to ensure the error-free execution of the whole change process and the accuracy of the information after the change [17]. Therefore, this paper establishes the service baselines for the on-site service period to achieve the purpose of retrospective changes.

### 2.3 Establishment of configuration baselines

Change Control is important to an effective CM program [16]. Change Control in CM emphasizes long-term tracking of changes to prevent unnecessary ones. However, a large number of changes in HME configuration will occur during the service life of the crane, forming a dynamic document database. The baseline technique is one of the management methods for Change Control, and it is also the basis for defining change. Major configuration baselines are known as the functional, allocated, and product baselines, as well as the developmental baseline [23]. However, in the face of changes in the HME configuration of the crane’s service life cycle, different baselines could be set according to specific requirements to achieve configuration continuity and traceability management [13].

**Fig 2:**
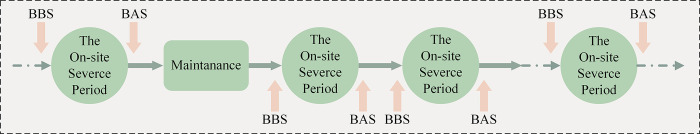
Logical relationship of change control between baselines.

The object of configuration control is the configuration baseline documents, and the basic concept of CM is to establish the baseline. The configuration baseline records the configuration of the target product at a specific time. It is a starting point and also a reference for subsequent activities [15]. However, the baseline management based on the HME configuration expands the scope of the original definition, which only focuses on the configuration of the product itself. It also extends the content of baseline records, with each baseline not only recording the configuration at the time of its formation, but also containing a record of the configuration at the time of the last baseline’s formation, and the configuration changes between these two baselines. With the occurrence of each service activity and maintenance activity, the resulting changes require configuration baselines to record and provide accurate data for subsequent activities [17]. Therefore, this paper takes the time point before and after each on-site service activity as the set point of the configuration baseline and establishes Baseline before Service (BBS), Baseline after Service (BAS). The logical relationship of Change Control between baselines is shown in Fig 2.

HME-CM’s baseline is created to manage changes better. Therefore, the data contained in it will not be all the HME configuration involved in the service life of the crane, but the configuration concerned with the construction and maintenance activities, such as information that will help avoid historical accidents, facilitate the traceability of accidents, and relate to changes in crane composition or part configuration. Baseline management is essentially a comparison management idea, which is to know the configuration changes by comparing the status at different time points [13]. However, the fact is that the HME configuration of the crane is always in a state of dynamic change. Therefore, the time points for setting up those baselines are discrete and determined according to the corresponding management requirements.

During the on-site service periods, all cranes will also undergo multiple detailed inspections or comprehensive inspections to ensure that they are in good condition and safe. The time point of inspections may occur after the modification of the lifting machinery and the occurrence of safety accidents, in addition to the replacement of the project or the site. Therefore, the end of the on-site service period and the construction of the BAS node also correspond to all the cases mentioned above and are not limited to the end of the project.

For configuration changes occurring during the in-plant maintenance period or on-site service period, no separate baseline will be established. All needed data related to changes in HME will be stored in the system as temporary change files. Those temporary files will be unified and summarized by BAS or BAM to form a comprehensive record, avoiding too many time nodes and affecting the efficiency of traceability queries. In addition to updating the previous baseline, all baselines are also a means of audit and inspection to avoid data registration errors and omissions and provide an accurate data basis for the next maintenance or service activity. Since Configuration Auditing is an important part of completing the CM process, all baselines need to be reviewed and confirmed by various stakeholders after establishment. If necessary, a review team will be jointly established by those stakeholders to ensure the rigor of the process [17].

### 2.4 The essence of the configuration baseline

**Fig 3:**
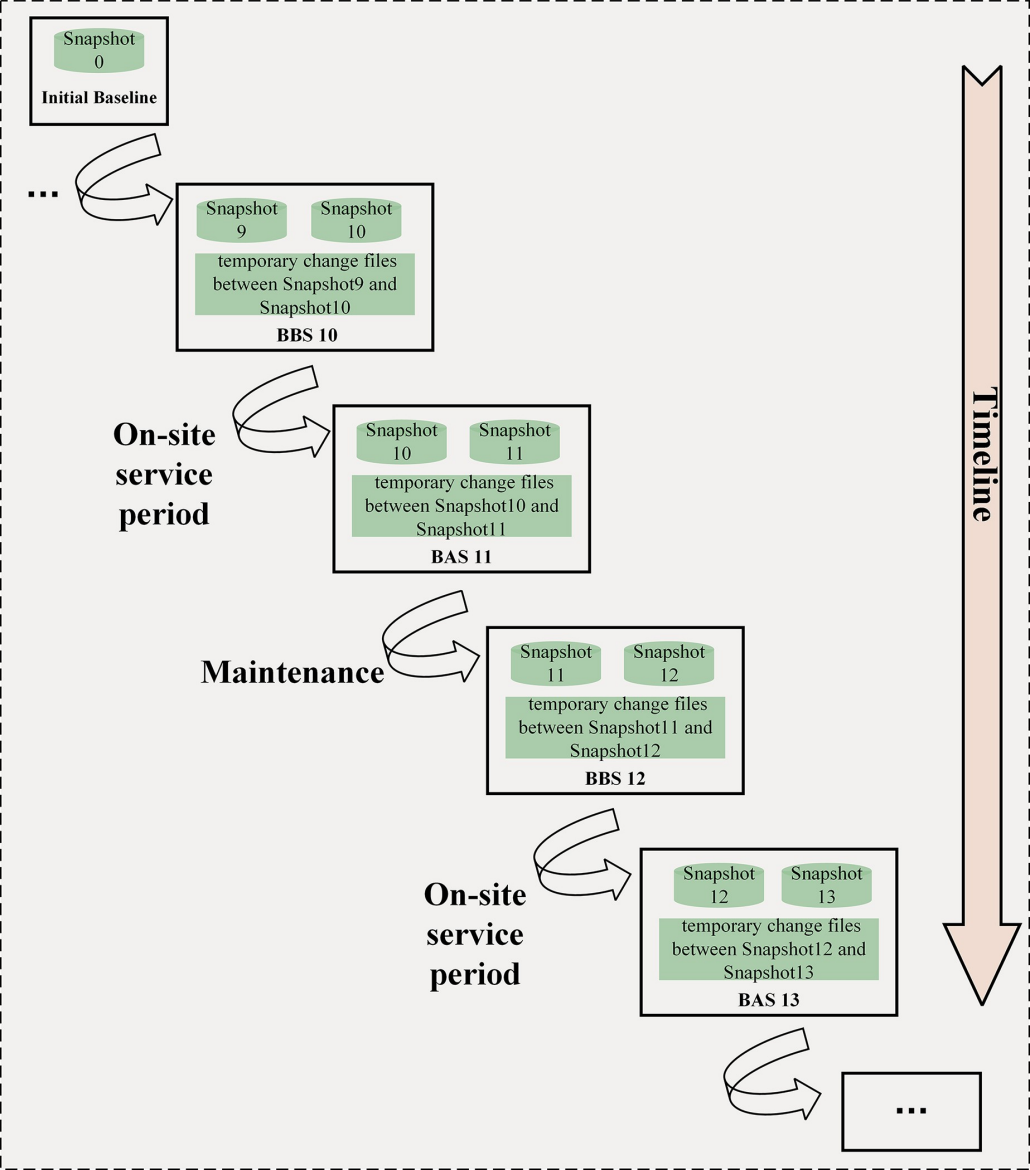
The essence of configuration baseline construction.

All configuration baselines are established along the timeline, and the current configuration is recorded when each baseline is formed, equivalent to a "snapshot," which captures the dynamic data at a specific point in time [18]. The baseline will record this snapshot and the snapshot formed at the previous baseline generation time, as well as the change information during the period. The details are shown in Fig 3. Those baselines can be used to control and create a lightweight and easily accessible database.

## 3. Content and updates to the HME configuration baseline

**Fig 4:**
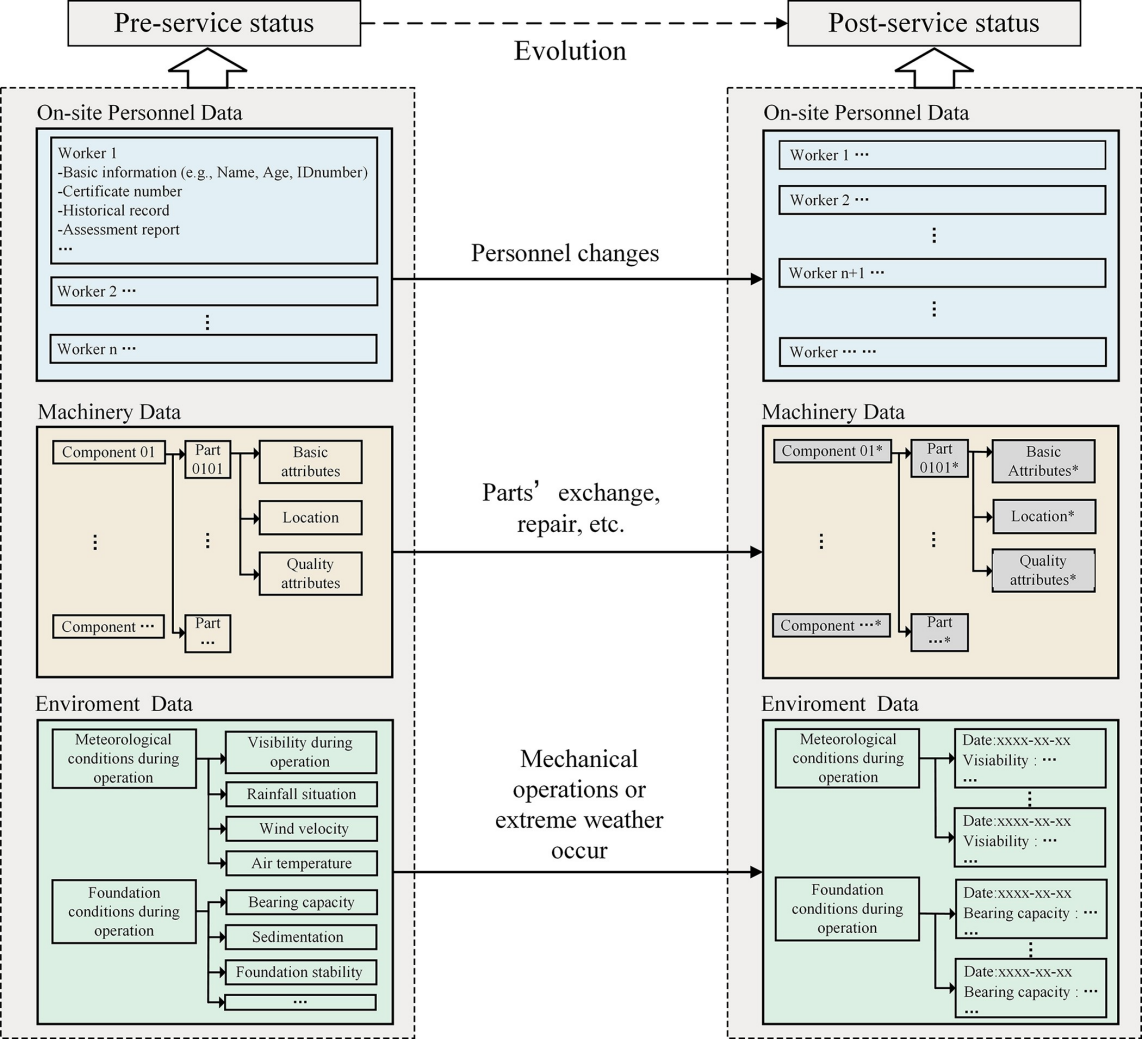
The typical evolution trajectory of HME configuration.

Fig 4 illustrates a typical evolving trajectory for the crane’s HME configuration during an on-site service period [24]. On-site human data is primarily concerned with personnel changes. Every person related to the crane must register and form temporary documents, such as operators, installation and disassembly workers, maintenance workers, and so on. Although most of the human factors that cause safety accidents are uncontrollable, there are also some of them that can be prevented by controlling and auditing workers’ information in advance, such as whether they are qualified and whether training is in place [11,25].

**Fig 5:**
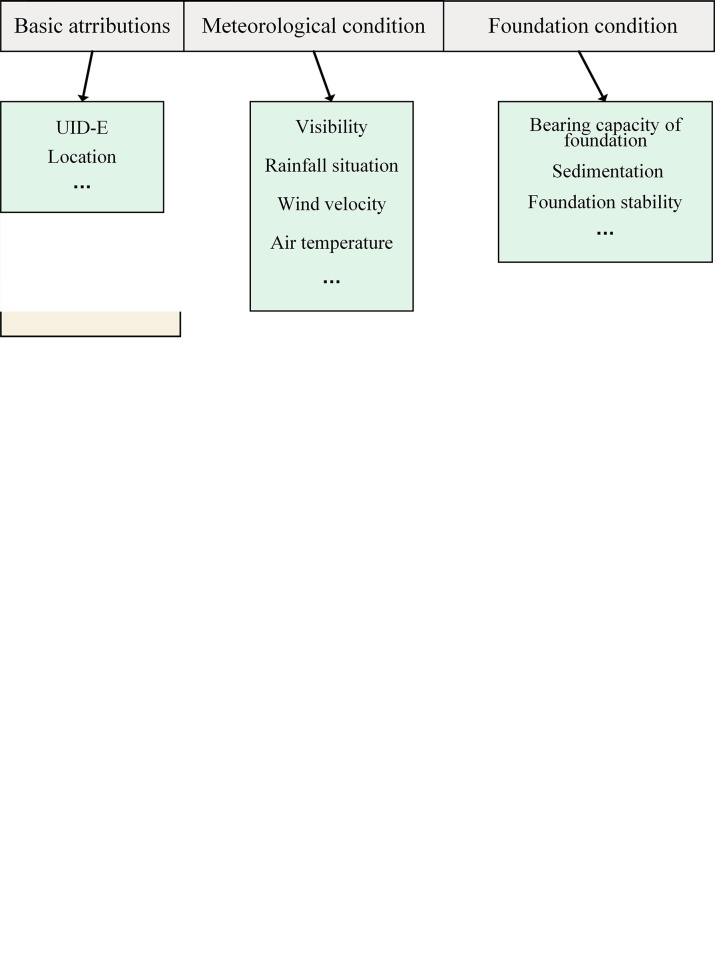
Configuration item of HME. (a) Human, (b) Machine, (c) Environment.

Therefore, as Fig 5(A) shows, the content to be updated of a personnel configuration item can be described by a structured quadruple as (Basic information, Certification, Historical record, Assessment), and each of them has detailed content. Here, the unique identifier (UID) is an important tool for finding temporary files generated by HME configuration changes [24]. There are three types of UID: UID-P, UID-M, and UID-E, which correspond to the human-machine-environment configuration in turn.

Mechanical configuration focuses on the state of parts. No matter what mechanical inspection and maintenance level is required, it is possible to trigger the repair, exchange, reuse, polish, or scrap of parts. Once the part undergoes the above changes, its configuration needs to be updated. As Fig 5(B) shows, the content to be updated of a mechanical configuration item can be described by a triple array of (Basic attributes, Location, Quality attributes).

Environmental data is primarily concerned with extreme weather and foundation conditions related to safety accidents. Meteorological and foundation conditions need to be recorded at each operation to ensure that they are suitable for the project’s unfolding. However, when extreme weather occurs during the service life cycle, it needs to be recorded even if the machinery is not used to facilitate accident accountability and analysis. Hence, as Fig 5(C) shows, the content to be updated of an environment configuration item can be described by a triple array as (Basic attributes, Meteorological condition, Foundation condition).

## 4. Marking of HME configuration

### 4.1 Basic concepts of data provenance

This paper intends to solve the problem of tracing the evolutionary history of HME configuration during a crane’s service life cycle by studying the data provenance of configuration documents.

Data provenance—sometimes called”data lineage” or “data pedigree”—refers to the evolutionary history of a piece of data, including its origin and all subsequent processing steps [26,27].

**Definition 1** (Data provenance). When data object A evolves into data object A’ after *n* subsequent processing processes, the data provenance (*DP*) of data object A’ from data object A can be expressed as initial data *InD* and subsequent processing *Ps*:

DP={InD,Ps} (1)

$$Ps=\{P_{1},P_{2},\ldots ,P_{n‐1},P_{n}\},n\;\epsilon \;N$$ (2)

Where *In* is the initial data of the data provenance, *P*_1_, *P*_2_,…, *P*_*n-*1_, *P*_*n*_ are the processes set after the source data, *P*_*i*_ is one of the processes, and *N* is the natural number set.

Using Eq (3), each data evolution *P*_*i*_ can be further detailed as follows:

$$\begin{array}{c} P_{i}=(In_{i},OP_{i},Out_{i}),i\;\epsilon \;N\\ In_{i}=(D_{i}{}_{1},\ldots ,D_{ik}),k\;\epsilon \;N\\ OP_{i}=(fi,TS_{i},TE_{i})\\ Out_{i}=(O_{i}{}_{1},\ldots ,O_{ij}),j\;\epsilon \;N \end{array}$$ (3)

where *In*_*i*_ is the set of input data for the process *P*_*i*_, *OP*_*i*_ is the set of specific operations during this process, and *Out*_*i*_ is the set of output data. Input and output data are *D*_*ik*_ and *O*_*ij*_ respectively. In addition, the dataset *OP*_*i*_ includes the operation attribute *f*_*i*_, start time *TS*_*i*_, and end time *TE*_*i*_ for every operation.

### 4.2 Modeling of the bill of HME configuration(BOHC)

In the third chapter, we clarified the object of HME-CM, where Fig 5 shows the three configuration items that make up the BOHC. Each configuration item is marked with a unique identifier (UID) during the service life and regarded as a node in BOHC. Among them, the UID-P for personnel is unique to each person, while the UID-E for the environment will not be used again once it is recorded. The usage rules for UID-M is more complicated than for UID-P and UID-E. After disassembling and scrapping a part from the machinery, its life cycle is terminated, and its information and UID-M will be archived and retained. When a part is remanufactured to become a new part, it will be assigned a new UID to initiate a new life cycle.

The BOHC of the crane is actually composed of these nodes. The mathematical model of the node set is as follows:

**Definition 2** (Node set of BOHC). Node set *C* is the set of all nodes *c* in BOHC:

$$\forall c\;\epsilon \;C,c=(UID_{c},a_{1},a_{2},\ldots ,a_{r}),r\;\epsilon \;N$$ (4)

Where *c* is a configuration item, UID_c_ is its unique code, *a*_1_, *a*_2_, …, *a*_*r*_ are the node attributes of this configuration item, the details of the node attributes are shown in Fig 5, and *r* is a natural number.

With the mathematical model of node set, the mathematical model of BOHC is defined as follows:

**Definition 3** (Snapshot of BOHC). The snapshot (*S*) of BOHC is the configuration of the object at a certain point in time. The BOHC model can be expressed as follows:

S=(C,T) (5)

Where (*C*, *T*) indicates this snapshot records the overall HME configuration at time point *T*.

### 4.3 Provenance annotation of BOHC

The process of obtaining data provenance, that is, data evolution history is data provenance tracking. The data specially stored for provenance tracing is called “provenance annotation” [26]. In order to achieve the purpose of marking and tracking HME configuration, we need to carry out provenance annotation design and BOHC data provenance modeling for them. First, we solve the problem of how to make the provenance annotations for those configuration items.

**Definition 4** (Data provenance of configuration item *c*). According to definition 1, the data provenance *DPc* of a configuration item *c* can be described as a set of initial data and subsequent processes.


$$\begin{array}{c} DP_{c}=\{(c,T_{0c}),Ps_{c}\},c\;\epsilon \;C\\ Ps_{c}=\{P_{0c},\ldots ,P_{ic},\ldots ,P_{nc}\},n\;\epsilon \;N\\ P_{ic}=(In_{ic},OP_{ic},Out_{ic}),i\;\epsilon \;N\\ In_{ic}=((c,TI_{ic}),\ldots )\\ OP_{ic}=(f_{ic},TS_{ic},TE_{ic})\\ Out_{ic}=((c,TO_{ic}),\ldots )\\ \left(TS_{ic},TE_{ic})\in (TI_{ic},TO_{ic}\right) \end{array}$$ (6)

From the above equations, the initial data of a configuration item can be expressed as (*c*, *T*_0*c*_). In addition, *D*_*ik*_ and *D*_*ij*_ in Eq (3) can be expressed as (*c*, *TI*_*ic*_) and (*c*, *TO*_*ic*_) in Eq (6). The operation attribute *f*_*ic*_ in Eq (6) is recorded differently for the 3 different configuration items: for personnel, *f*_*ic*_ is their work record; for machinery, *f*_*ic*_ is their maintenance process record; and for the environment, *f*_*ic*_ is the condition record for a certain time.

During the whole service life cycle of the crane, the provenance annotation *Pa* of the configuration item will be stored additionally to record those changes.

**Definition 5** (Provenance annotation of configuration item *c*). Use ${Pa}_{{UID}_{c}}$ as the provenance annotation of a configuration item *c* and record its one-time process:

$$Pa_{UID_{c}-1}=(ty,UID_{c},P_{ic}‐UID_{c},TI_{ic},TO_{ic})$$ (7)

In order to facilitate the subsequent traceability steps, *ty* in Eq (7) indicates the specific type of its UID, and *P*_*ic*_*-*UI*D*_*c*_ specifies which process of this configuration item this is. Taking the personnel configuration item *c*_1_ as an example, assuming that its UID is 10001 and its 2nd fieldwork is from July 1, 2022, to August 1, 2022, its corresponding provenance annotation is:

$$Pa_{10001‐002}=(H,10001,P_{00}{}_{2}‐10001,2021.7.1,2021.8.1)$$ (8)

**Definition 6** (Set of provenance annotations for configuration item). With ${LPa}_{{UID}_{c}}$ marking the provenance annotation of configuration item *c* and ${Pa}_{{UID}_{c}-v}$ marking a provenance annotation record, ${LPa}_{{UID}_{c}}$ is the set of all ${Pa}_{{UID}_{c}}$ of configuration item *c*:

$$LPa_{UID_{c}}=(Pa_{UID‐0},Pa_{UID‐1},Pa_{UID‐3},\ldots ,Pa_{UID‐v}),v\;\epsilon \;N$$ (9)

### 4.4 BOHC data provenance model based on provenance annotation

The BOHC data provenance model is essentially a baseline data provenance model built on snapshots. Set the snapshot of the crane that needs to track the provenance as *Q*_*n*_, then the initial snapshot is *Q*_0_, and the subsequent snapshots are *Q*_1_, …,*Q*_*n*-1_. Therefore, one snapshot of crane *X* is recorded as $X_{Q_{i}}$, and all the configuration items related to crane *X* will be recorded each time the snapshot is generated. According to Eq (6):

$$\forall X\in C,\;X_{Q_{i}}=(X,T_{Q_{i}})$$ (10)

Then the data provenance model for BOHC of crane *X* is:

$$\begin{array}{c} DP_{Q_{n}}=\{X_{Q_{0}},Ps_{Q_{n}}\}\\ Ps_{Q_{n}^{}}=(P_{Q_{0}},P_{Q_{1}},\ldots ,P_{Q_{n‐1}}),n\;\epsilon \;N \end{array}$$ (11)

One processing step $P_{Q_{i}}$ of HME configuration is to record the two snapshots at the beginning and end of this step, as well as the changes between them; in other words, the data recorded by $P_{Q_{i}}$ is the information present in the baseline which is generated at the end time $TE_{Q_{i}}$. The input data is $X_{Q_{i}}$, and the output data is the new version of HME configuration $X_{Q_{i+1}}$, which can be expressed as:

$$\begin{array}{c} P_{Q_{i}}=(In_{Qi},OP_{Q_{i}},Out_{Qi})\\ In_{Q_{i}}=(X,TI_{Q_{i}})=((c,TI_{Q_{i}}),\ldots )\\ OP_{Q_{i}}=(LPa_{Q_{i}},TS_{Q_{i}},TE_{Q_{i}})\\ Out_{Q_{i}}=(X,TO_{Q_{i+1}})=((c,TO_{Q_{i+1}}),\ldots ) \end{array}$$ (12)

Where $OP_{Q_{i}}$ indicates the changes between snapshots $X_{Q_{i}}$ and $X_{Q_{i+1}}$, specifically records all provenance annotations in the time ($TS_{Q_{i}},TE_{Qi}$), and forms the provenance annotation set $LPa_{Q_{i}}$.

## 5. Search procedures for HME configuration’s tracking and tracing

**Fig 6:**
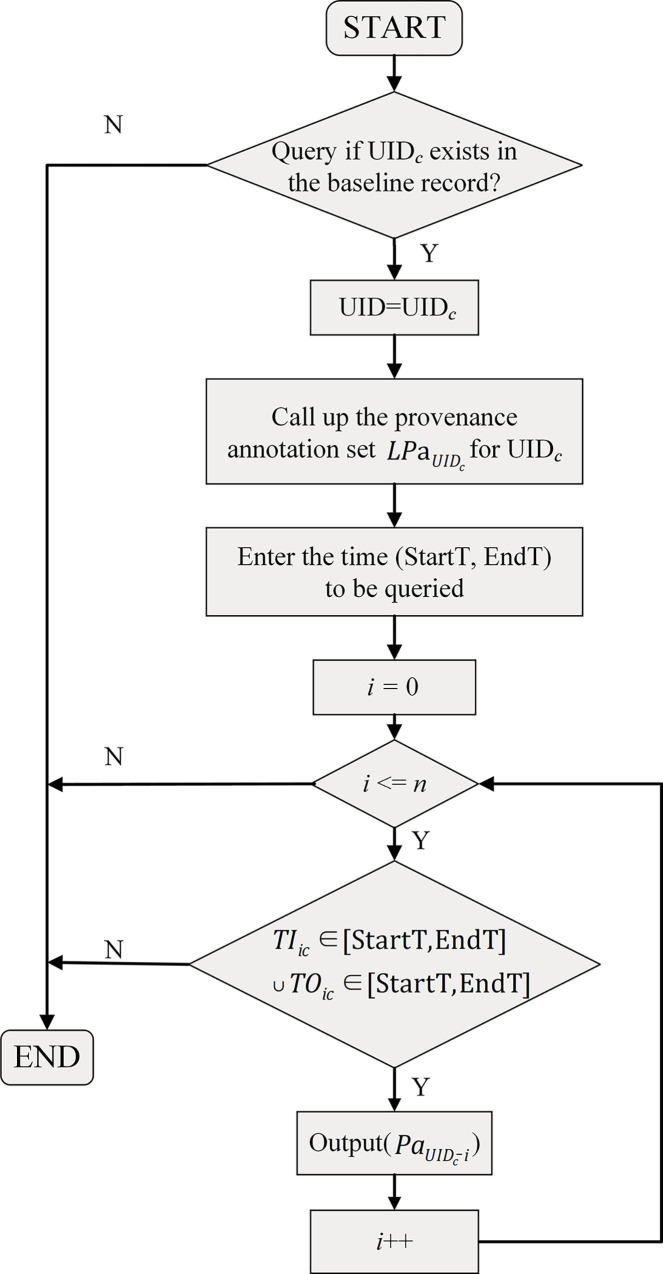
Search procedures (a).

The HME configuration’s tracking and tracing are essential parts of HME-CM. Throughout the service life cycle, there will be countless times when the historical configuration needs to be checked. Especially when a quality issue occurs, the problem-solving efficiency relies on the HME configuration’s tracking and tracing abilities [24]. Typically, there are two categories of requirements. One aims to query the configuration of a configuration item for a specific time along the timeline. The other is in the case where there is no specific query object but only a time point or time to be queried. Based on the UID of the configuration item and the configuration baseline, the corresponding search procedures can be designed to meet both types of requirements as follows:

The first situation aims at tracking specific configuration items along the timeline. The input data at this point are the UID of the configuration item c and the query time, which we assume to be UID_c_ and (StartT, EndT). The output data is the provenance annotation of the configuration item for that time to facilitate the subsequent query of detailed information. The procedure is depicted in Fig 6. In this procedure, when it is determined that UID_c_ exists in the baseline, all provenance annotations for UID_c_ are called up for query purposes. We start with the earliest provenance annotation and determine whether it is within (StartT, EndT) by its start time *TI*_*ic*_ and end time *TO*_*ic*_, and output the provenance annotation ${Pa}_{{UID}_{c}}$ if it is confirmed to be within the time.

**Fig 7:**
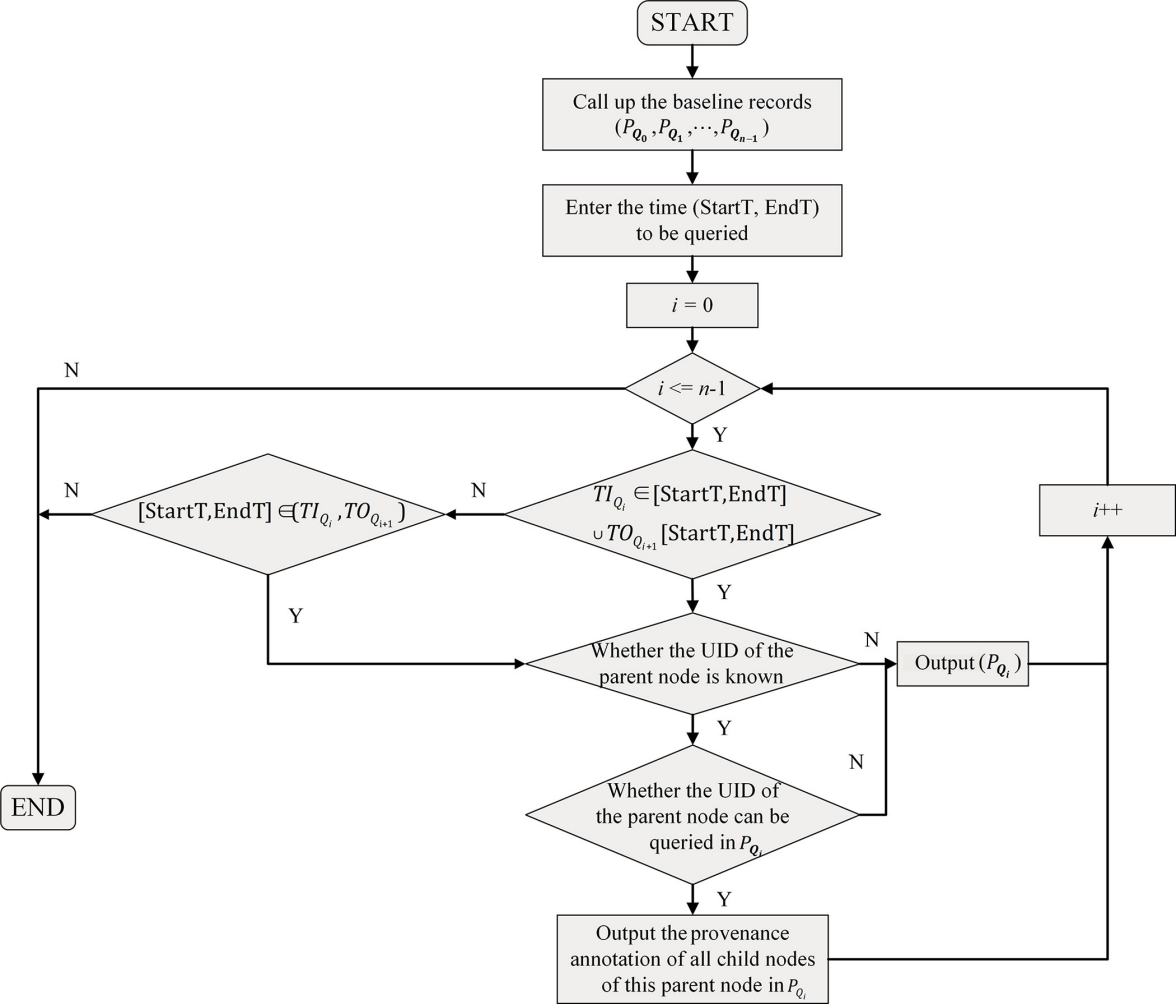
Search procedures (b).

The second case aims at dealing with the situation when we do not know which configuration item we need to query. The input data in this case is the time (StartT, EndT). The procedure is depicted in Fig 7. First, we call up the baseline records, starting from the earlier baseline, and if the start time *TI*_*Q*_ or end time *TO*_*Q*_ of the baseline is within (StartT, EndT), we output the baseline data *P*_*Q*_. But there is a special case here. When it comes to the mechanical configuration item, although we can not know the specific UID, it is possible to tell which its parent node is. In this case, we will output the provenance annotations of all the child nodes of that parent node.

## 6. Application example

**Fig 8:**
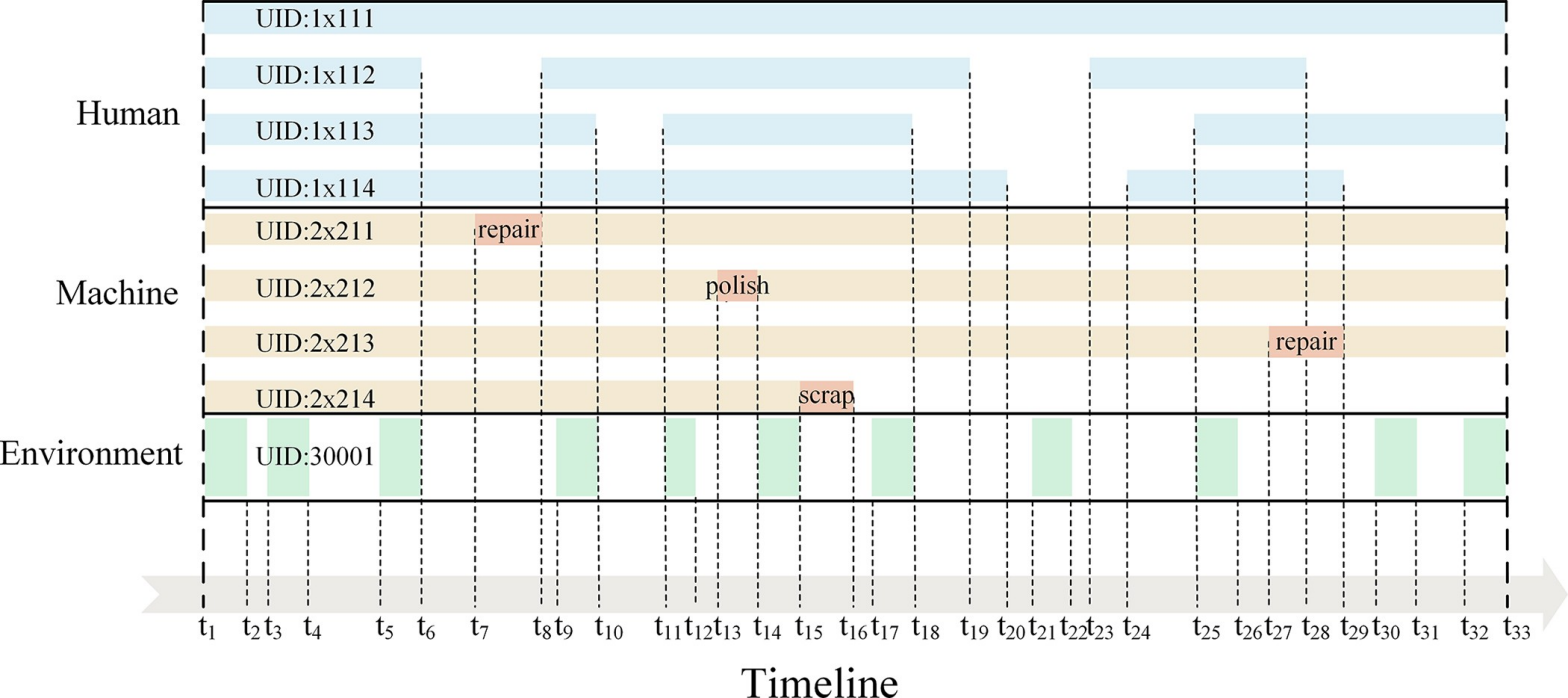
Configuration item change activities of T19xx-0001.

In the plural, complex, and uncertain project-based construction environments [28], the safety of cranes is a complex socio-technical system [8], which can be influenced by a variety of factors across multiple dimensions, including human, machine, and environmental factors [29]. The data related to these factors is massive and in a state of frequent change. Also, tracking and tracing data change records for a crane is not a simple task within the construction industry. Over time, those data may be lost, destroyed, or not even recorded [7]. Therefore, an immutable, accurate, timely, and traceable way is needed to help safety managers record important information in order to allow for the timely identification of problems, unearth the context within which an accident arises, enable accountability to be shared across different stakeholders, and facilitate continuous improvement in safety management [7,14]. To solve this problem, we proposed the HME-CM approach that can disclose the history quickly and clearly, and the following is an application example:

The amount of HME data involved in a crane’s service life is massive. Therefore, to simplify the data volume and better illustrate the feasibility of this approach, we take the crane numbered T19xx-0001 as an example and list some of the lowest-level configuration item change activities to show the management and control of HME configuration. The data information is shown in Fig 8 and Table 1, and the detailed information on configuration items such as attributes is not expanded here. Meanwhile, the baselines before and after those change activities are BBS 7 and BAS 8, generated at time points t_S_ and t_E_, respectively. The provenance annotations generated by those change activities are shown in Table 2, and the corresponding information recorded by baseline BAS 8 is shown in Table 3.

**Table 1:** Information on the configuration item change activities of T19xx-0001.

Type of configuration item	UID	Configuration evolution	Time	Process order
H	1x111	Field operation	(t_1_, t_33_)	xx1
	1x112	Field operation	(t_1_, t_6_)	xx2
		Field operation	(t_9_, t_19_)	xx3
		Field operation	(t_23_, t_28_)	xx4
	1x113	Field operation	(t_1_, t_10_)	xx5
		Field operation	(t_11_, t_18_)	xx6
		Field operation	(t_25_, t_33_)	xx7
	1x114	Field operation	(t_1_, t_20_)	xx8
		Field operation	(t_24_, t_29_)	xx9
M	2x211	Repair	(t_7_, t_8_)	x10
	2x212	Polish	(t_13_, t_14_)	x11
	2x213	Repair	(t_27_, t_29_)	x12
	2x213	Scrap	(t_15_, t_16_)	x13
E	30001	Mechanical operation	(t_1_, t_2_)	x14
		Extreme weather	(t_3_, t_4_)	x15
		Mechanical operation	(t_5_, t_6_)	x16
		Mechanical operation	(t_9_, t_10_)	x17
		Mechanical operation	(t_11_, t_12_)	x18
		Extreme weather	(t_14_, t_15_)	x19
		Mechanical operation	(t_17_, t_18_)	x20
		Extreme weather	(t_21_, t_22_)	x21
		Mechanical operation	(t_25_, t_26_)	x22
		Mechanical operation	(t_30_, t_31_)	x23
		Mechanical operation	(t_32_, t_33_)	x24

**Table 2:** Provenance annotations of the change activities.

UID	Time	Process order	Provenance annotation (*Pa*)
1x111	(t_1_, t_33_)	x11	*Pa*_1x111-x11_ = (H, 1x111, *p*_x11_-1x111, t_1_, t_33_)
1x112	(t_1_, t_6_)	x12	*Pa*_1x112-x12_ = (H, 1x112, *p*_x12_-1x112, t_1_, t_6_)
	(t_9_, t_19_)	x13	*Pa*_1x112-x13_ = (H, 1x112, *p*_x13_-1x112, t_9_, t_19_)
	(t_23_, t_28_)	x14	*Pa*_1x112-x14_ = (H, 1x112, *p*_x14_-1x112, t_23_, t_28_)
1x113	(t_1_, t_10_)	x15	*Pa*_1x113-x15_ = (H, 1x113, *p*_x15_-1x113, t_1_, t_10_)
	(t_11_, t_18_)	x16	*Pa*_1x113-x16_ = (H, 1x113, *p*_x16_-1x113, t_11_, t_18_)
	(t_25_, t_33_)	x17	*Pa*_1x113-x17_ = (H, 1x113, *p*_x17_-1x113, t_25_, t_33_)
1x114	(t_1_, t_20_)	x18	*Pa*_1x114-018_ = (H, 1x114, *p*_x18_-1x114, t_1_, t_20_)
	(t_24_, t_29_)	x19	*Pa*_1x114-019_ = (H, 1x114, *p*_x19_-1x114, t_24_, t_29_)
2x211	(t_7_, t_8_)	x21	*Pa*_2x211-x21_ = (M, 2x211, *p*_x21_-2x211, t_7_, t_8_)
2x212	(t_13_, t_14_)	x22	*Pa*_2x212-x22_ = (M, 2x212, *p*_x22_-2x212, t_13_, t_14_)
2x213	(t_27_, t_29_)	x23	*Pa*_2x213-x23_ = (M, 2x213, *p*_x23_-2x213, t_27_, t_29_)
2x214	(t_15_, t_16_)	x24	*Pa*_2x214-x24_ = (M, 2x214, *p*_x24_-2x214, t_15_, t_16_)
30001	(t_1_, t_2_)	x31	*Pa*_30001-x31_ = (E, 30001, *p*_x31_-30001, t_1_, t_2_)
	(t_3_, t_4_)	x32	*Pa*_30001-x32_ = (E, 30001, *p*_x32_-30001, t_3_, t_4_)
	(t_5_, t_6_)	x33	*Pa*_30001-x33_ = (E, 30001, *p*_x33_-30001, t_5_, t_6_)
	(t_9_, t_10_)	x34	*Pa*_30001-x34_ = (E, 30001, *p*_x34_-30001, t_9_, t_10_)
	(t_11_, t_12_)	x35	*Pa*_30001-x35_ = (E, 30001, *p*_x35_-30001, t_11_, t_12_)
	(t_14_, t_15_)	x36	*Pa*_30001-x36_ = (E, 30001, *p*_x36_-30001, t_14_, t_15_)
	(t_17_, t_18_)	x37	*Pa*_30001-x37_ = (E, 30001, *p*_x37_-30001, t_17_, t_18_)
	(t_21_, t_22_)	x38	*Pa*_30001-x38_ = (E, 30001, *p*_x38_-30001, t_21_, t_22_)
	(t_25_, t_26_)	x39	*Pa*_30001-x39_ = (E, 30001, *p*_x39_-30001, t_25_, t_26_)
	(t_30_, t_31_)	x40	*Pa*_30001-x40_ = (E, 30001, *p*_x40_-30001, t_30_, t_31_)
	(t_32_, t_33_)	x41	*Pa*_30001-x41_ = (E, 30001, *p*_x41_-30001, t_32_, t_33_)

**Table 3:** Information on the BAS 8.

Baseline	Information present in the baseline		
BAS 8	Snapshot 7	UID	Attributes ($TI_{Q_{7}}$ = t_S_)
		…	…
		1x111	*a*_1_, *a*_2_, …, *a*_*r*_
		1x112	*a*_1_, *a*_2_, …, *a*_*r*_
		…	…
		2x211	*a*_1_, *a*_2_, …, *a*_*r*_
		2x212	*a*_1_, *a*_2_, …, *a*_*r*_
		…	…
		30001	*a*_1_, *a*_2_, …, *a*_*r*_
		30001	*a*_1_, *a*_2_, …, *a*_*r*_
		…	…
	Temporary change files	UID	Provenance annotation (*Pa*)
		…	…
		1x111	(H, 1x111, *p*_x11_-1x111, t_1_, t_33_)
		1x112	(H, 1x112, *p*_x12_-1x112, t_1_, t_6_)
		…	…
		2x211	(M, 2x211, *p*_x21_-2x211, t_7_, t_8_)
		2x212	(M, 2x212, *p*_x22_-2x212, t_13_, t_14_)
		…	…
		30001	(E, 30001, *p*_x31_-30001, t_1_, t_2_)
		30001	(E, 30001, *p*_x32_-30001, t_3_, t_4_)
		…	…
	Snapshot 8	UID	Attributes ($TO_{Q_{8}}$ = t_E_)
		…	…
		1x111	*a*_1_, *a*_2_, …, *a*_*r*_
		1x112	*a*_1_, *a*_2_, …, *a*_*r*_
		…	…
		2x211	*a*_1_, *a*_2_, …, *a*_*r*_
		2x212	*a*_1_, *a*_2_, …, *a*_*r*_
		…	…
		30001	*a*_1_, *a*_2_, …, *a*_*r*_
		30001	*a*_1_, *a*_2_, …, *a*_*r*_
		…	…

**Fig 9:**
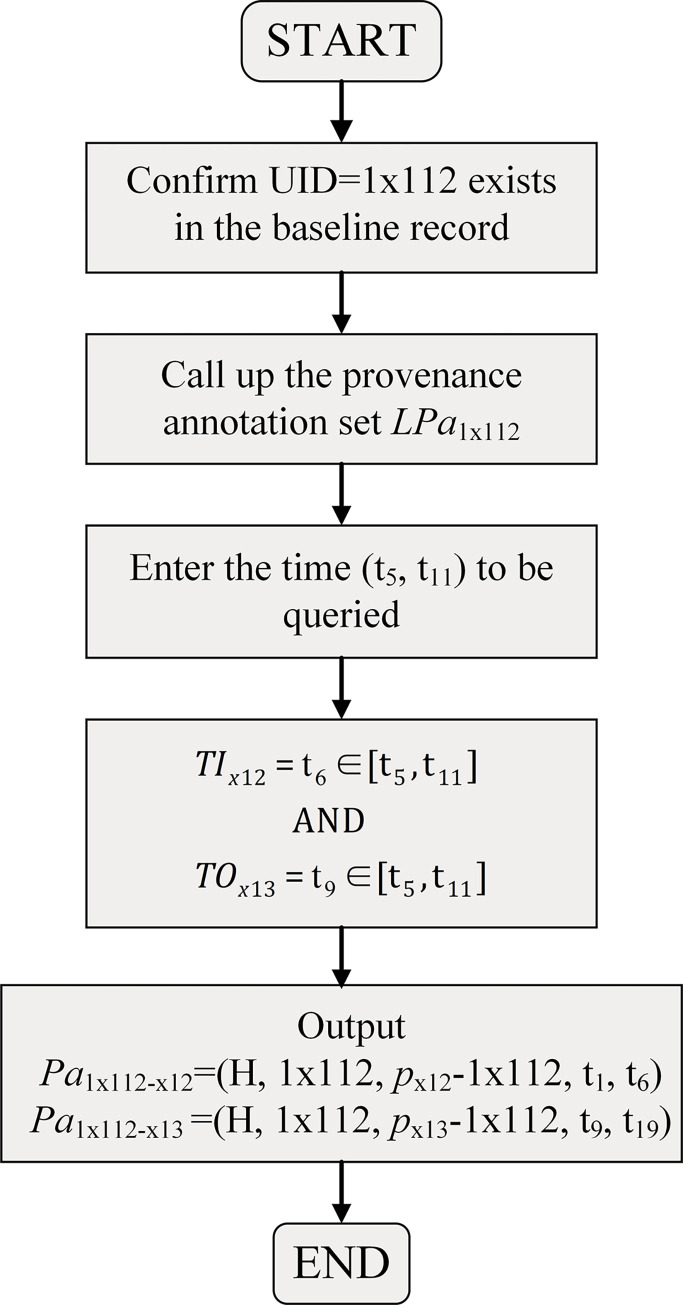
Tracing steps (a).

Based on the information listed above, when we query the configuration of a configuration item for a specific time along the timeline, assuming that the UID of the configuration item is 1x112 and the query time is (t_5_, t_11_), the specific steps are shown in Fig 9.

**Fig 10:**
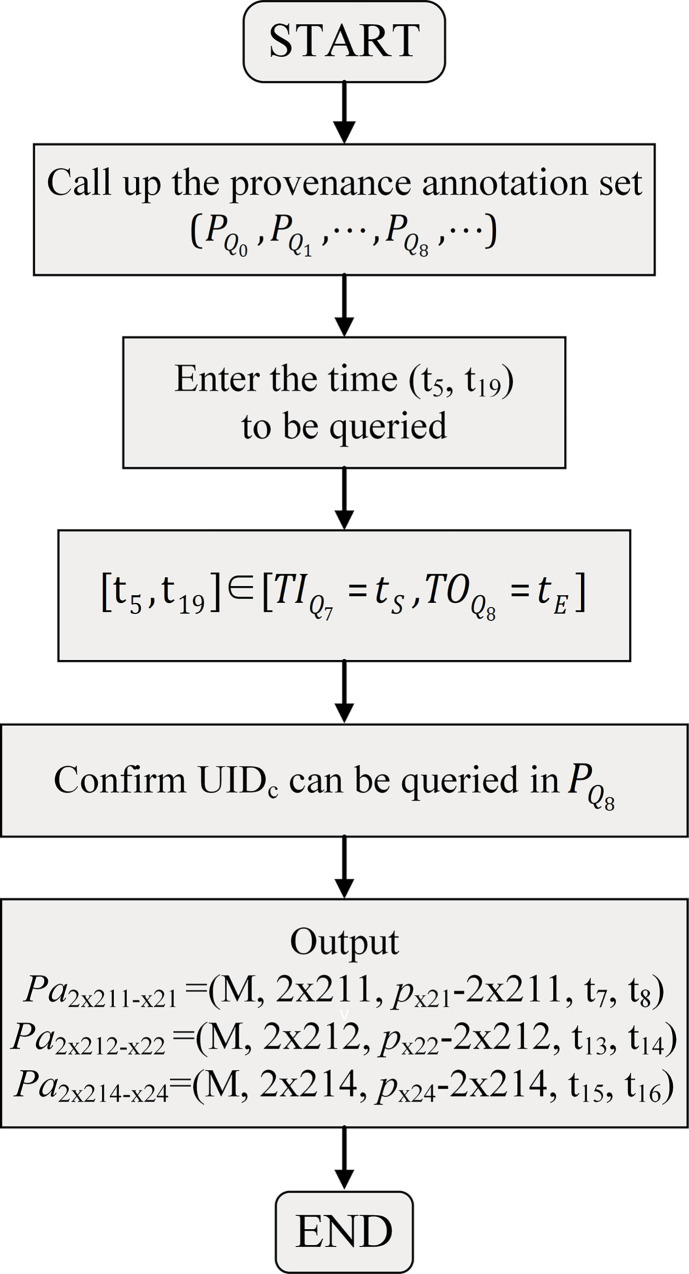
Tracing steps (b).

When we do not know which configuration item needs to be queried but only its parent node and period, suppose the time we need to query is (t_5_, t_19_), and the UID of the parent node of configuration items 2x211, 2x212, 2x213, and 2x214 is UID_c_, then the specific steps are shown in Fig 10.

As can be seen from the above example, the HME-CM approach is effective in recording every configuration item’s evolving trajectory for the crane. Based on the use of provenance annotation, both change management and tracking and tracing realization are implemented. And with the standardization of the HME-CM process, the omission of important information is controlled, and quick retrieval of data is facilitated. In this way, a transparent, accurate, and traceable database is provided to inform managerial decision-making.

## 7. Discussion

This paper applied CM theory to study the safety management method of the crane during the service life cycle from the perspective of HME. Based on the three elements (HME) that have the deepest impact on crane safety, we have expanded the scope of CM and the definition of baseline to make CM more applicable to the safety management of the crane. Moreover, for the critical marking process in CM, the BOHC data provenance model was built, and two searching procedures were proposed so that the HME configuration can be easily tracked and traced.

Based on the BOHC data provenance model and search procedures, we can solve the problem of too much storage of HME configuration data and make the HME-CM process standardized. Various data related to construction safety are managed in configuration identification, which is crucial for construction safety but lacks research [13]. Therefore, we newly defined baselines in CM and proposed an easily managed marking method, which not only makes configuration changes identifiable and facilitates subsequent queries but also allows auditors to detect non-conformances in time, increasing the reliability of crane construction.

As the first study to use CM theory to manage the HME elements that affect crane safety, some limitations are in this paper. The change contents of concern for the three kinds of configuration items are summarized from the previous literature, but they may still be incomplete. However, in the specific implementation process, the content of concern can be added or subtracted according to the project experience and the specific conditions to make the management more reliable. Moreover, it is worth noting that we have only proposed two search procedures in the paper and that there may be more efficient ways or different search requirements, after which we will work on these aspects.

## 8. Conclusions

This paper presents the process of CM applications to manage crane safety. Specifically, we explored how to conduct configuration change management from the HME perspective of the crane. To build the HME-CM framework, based on the analysis of the factors affecting crane safety, we specified the management objects, which are the three kinds of configuration items, and explored their attributes that need to be of interest when the configuration is changed. After that, the concept of BOHC was proposed, and the provenance annotation method was applied to construct the BOHC data provenance model, which is the basis for tracking and tracing the evolution history of HME. Finally, some search procedures were presented to fulfill the CM requirements in the crane’s service life cycle.

The complexity of the machines, the diversity of the data, and the dynamic nature of HME configuration all add to the difficulty of managing safety. An effective managerial method is the key to solving these problems. The HME-CM approach based on CM theory can realize data controllability, continuity, and consistency. In the future, CM for the crane can also be combined with digital twin technology and blockchain technology to achieve immutable, transparent, and efficient information recording [30].
